# Supplementary material for: Environmentally-induced parental or developmental conditioning influences coral offspring ecological performance
Source: Sci Rep. 2020 Aug 12;10:13664. doi: 10.1038/s41598-020-70605-x (PMC7423898; doi:10.1038/s41598-020-70605-x)
Supplement: Supplementary file 1 — Supplementary Information [file 41598_2020_70605_MOESM1_ESM.docx]

**Environmentally-induced parental or developmental conditioning influences coral offspring ecological performance**

Hollie M. Putnam^1^, Raphael Ritson-Williams^2^, Jolly Ann Cruz^3^, Jennifer M. Davidson^2^, Ruth D. Gates^2^

^1^University of Rhode Island, Department of Biological Sciences, Kingston, RI USA

^2^Hawai‘i Institute of Marine Biology, University of Hawai‘i, Mānoa, HI, USA

^3^Micronesia Islands Nature Alliance, Garapan, Saipan 96950, CNMI

* Corresponding author: hputnam@uri.edu, 401-874-9510

**Supporting Information**

**Figure S1. Experimental Conditions.** Temperature (°C), PAR (µmol photons m^-2^ s^-1^), and NBS pH measured every 15 minutes for the periods of adult acclimation, adult exposure, larvae 1 month, and larval 6 months. See methods for full measurement details. Figures show mean ± sem for data with 15min frequency for a mean daily cycle calculated from each period. No pH data are available for acclimation period.

**Table S1.** **Statistical results from GLMM analysis** of survivorship, settlement, and growth rate (log transformed). Tile sample sizes for survivorship and settlement time 0, month 1 and month 6 and growth at month 1 were Ambient origin, Ambient offspring = 36, High origin, Ambient offspring = 12, Ambient origin, High offspring = 36, and High origin, High offspring = 12. Tile sample sizes for growth rate at month 6 were Ambient origin, Ambient offspring = 26, High origin, Ambient offspring = 10 Ambient origin, High offspring = 21, and High origin, High offspring = 7.

***Survivorship of offspring at time 0, and spat at months 1 and 6***

| **Factor** | **Df** | **Chi-Square** | **p** |
| --- | --- | --- | --- |
| *Origin* | 1 | 5.754 | ***0.016*** |
| *Secondary* | 1 | 32.747 | ***<0.0001*** |
| *Time* | 2 | 879.012 | ***<0.0001*** |
| *Secondary * Time* | 2 | 9.583 | ***0.008*** |

***Settlement***

| **Factor** | **Df** | **Chi-Square** | **p** |
| --- | --- | --- | --- |
| *Origin* | 1 | 5.306 | ***0.021*** |
| *Secondary* | 1 | 27.38 | ***<0.0001*** |

***Growth Rate***

| **Factor** | **numDF** | **denDF** | **F value** | **p** |
| --- | --- | --- | --- | --- |
| *Origin* | 1 | 94 | 4.778 | ***0.031*** |
| *Time* | 1 | 94 | 258.627 | ***<0.0001*** |
| *Origin * Time* | 1 | 62 | 3.792 | 0.056 |
